# A case of enterocutaneous fistula treated with a dome-shaped negative-pressure irrigation and drainage device

**DOI:** 10.1097/MD.0000000000047047

**Published:** 2026-01-09

**Authors:** Jun Gao, Hao Sun, Xiyan Qin, Zhide Sun

**Affiliations:** a Emergency Department, Affiliated Hospital of Chengde Medical University, Chengde, Hebei Province, China; b Medical Materials Department, Affiliated Hospital of Chengde Medical University, Chengde, Hebei Province, China.

**Keywords:** enterocutaneous fistula, irrigation and drainage, limiting spillage, negative-pressure device, visualization

## Abstract

**Rationale:**

Enterocutaneous fistula (ECF) is a severe post-gastrointestinal surgery complication with low cure rates, prolonged treatment cycles, and a global mortality rate of 20% to 44%. Currently, common clinical non-surgical treatments have issues like fluid leakage and inadequate drainage, while existing negative-pressure suction dressings hinder fistula opening observation. Thus, exploring a new therapeutic device integrating “effective drainage, leakage-proof protection, and visual observation” is highly significant for improving ECF patients’ prognosis.

**Patient concerns:**

This article describes a case of a 66-year-old male patient. The patient underwent right hemicolectomy for intestinal obstruction and colon cancer, then emergency laparotomy for hemostasis due to acute intra-abdominal active bleeding postoperatively. On postoperative day 6, abdominal incision dehiscence occurred and was managed with debridement and suturing. Five days after suture, massive digestive fluid leakage (up to 2500 mL daily) was noted from the incision, with a 0.5 × 0.5 cm enterocutaneous fistula identified at the incision base. Abdominal computed tomography showed the fistula was located in the lower part of the incision. Diagnosis: Enterocutaneous fistula.

**Diagnoses:**

The patient was diagnosed with an enterocutaneous fistula within the incision and a large amount of digestive fluid leakage.

**Interventions:**

The patient was treated with a dome-shaped negative-pressure irrigation and drainage device covering the enterocutaneous fistula orifice and incision, with continuous negative-pressure irrigation and drainage. Concurrently, adjunctive treatments were given, including anti-infection therapy, parenteral nutritional support, and digestive fluid secretion inhibition.

**Outcomes:**

After 11 days of treatment with a dome-shaped negative-pressure irrigation and drainage device, the patient’s enterocutaneous fistula healed, with fresh granulation tissue in the incision, a significant reduction in the incision area, and normalization of inflammatory markers.

**Lessons:**

In the acute and stable stages of enterocutaneous fistula (ECF), effective fistula drainage is key to promoting fistula healing. The dome-type negative-pressure irrigation and drainage device can enhance drainage efficacy, improve perifistular tissue cleanliness, boost granulation tissue growth, reduce dressing changes, and shorten fistula healing time. This paper aims to demonstrate the feasibility of using this device to promote ECF healing.

## 1. Introduction

An enterocutaneous fistula occurs in the gastrointestinal tract through the skin or wounds and the formation of abnormal channels outside the body. After the formation of an enterocutaneous fistula, a large amount of digestive fluid rich in digestive enzymes as well as feces and pus leak out from the leakage port, corroding the tissue around the fistula and causing the patient’s local tissues to become red, swollen, vesicose, painful, and uncomfortable.^[1]^ It is a consistently serious complication after abdominal surgery due to its association with the risk of water and electrolyte disturbances, nutrient loss, soft tissue infection of the abdominal wall and necrotizing fasciitis, and systemic infections, and has a 20% to 44% mortality rate^[2]^, current non-surgical treatment strategies for enterocutaneous fistulas include infection control, nutritional support, and maintenance of cleanliness around the fistula to promote self-healing of the leak.^[3]^ Adequate and effective drainage is essential to clean the skin and soft tissues around the fistula. Currently, the commonly used treatment methods include simple dressing change, ostomy bag covering the fistula, drip double trocar method, negative-pressure suction, and negative-pressure suction combined with flushing,^[4–6]^ however, these are still unable to solve the problem of spillage and skin corrosion when the flow rate of the leakage liquid increases. Therefore, we utilized existing medical materials to create a dome-shaped negative-pressure irrigation and drainage device for the treatment of enterocutaneous fistula and discussed its therapeutic effect in 1 case.

## 2. Case presentation

### 2.1. General information

**Figure 1.**
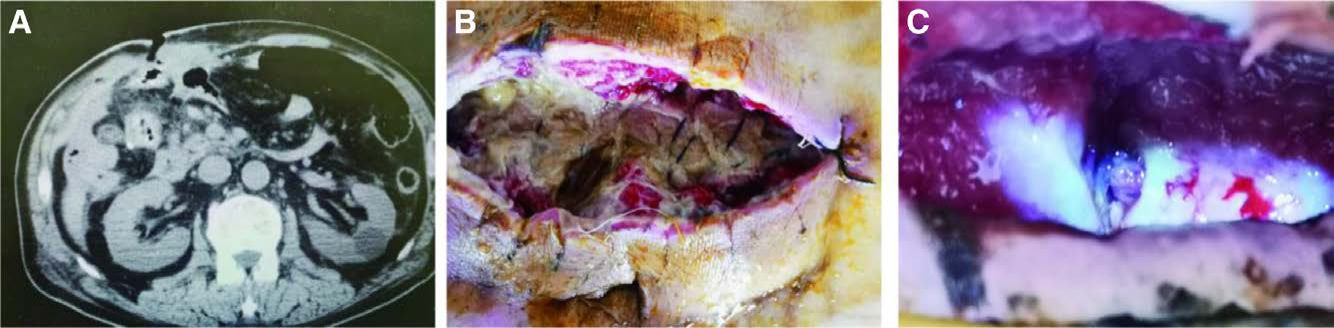
CT images and incision changes. (A) enterocutaneous fistula occurs with a distinct sinus tract, no abscesses seen. (B) Incision split with necrotic tissue attached to the base. (C) enterocutaneous fistula at the base of the incision, with leakage of digestive fluids. CT = computed tomography.

A 66-year-old male admitted to the hospital with abdominal pain and distension, nausea and vomiting, cessation of gas, and defecation for 3 days. Past history of chronic gastritis, esophagitis, duodenal bulbitis. The patient was admitted with the following diagnoses: intestinal obstruction, malignant tumor of the colon. After admission, radical resection of the right hemicolectomy was performed, postoperative active abdominal bleeding occurred, and open abdominal exploration for hemostasis was performed under emergency general anesthesia. On the 6th postoperative day, the patient developed an abdominal wall incision split, and another abdominal wall incision split debridement suture was performed, and a large amount of digestive fluid leakage was seen in the incision at the 11th postoperative day, up to 2500 mL per day, remove the suture, at the bottom of the incision, the fistula of enterocutaneous fistula can be seen, the size is about 0.5 × 0.5 cm, complicated by enterocutaneous fistula, abdominal computed tomography suggests that the fistula is located in the lower part of the incision, adjacent to the incision, and the surrounding tissues form an adhesion, and the formation of abscess around the incision and the abdominal cavity does not see any clear formation of abscess (Fig. 1).

### 2.2. Treatment

Patients with complications of enterocutaneous fistula were given fasting water, gastrointestinal decompression, anti-infection, nutritional support, and other basic treatment to correct water, electrolyte, and acid-base balance disorders; at the same time, the application of dome-shaped negative-pressure irrigation and drainage device to cover the enterocutaneous fistula mouth and incision, according to the size of the leakage of liquid to adjust the flushing flow rate and the size of the negative pressure, to maintain the fistula tract clean, protect the skin around the fistula mouth to prevent the corrosion of digestive fluids; every 2 days to replace the once.

### 2.3. Treatment results

**Figure 2.**
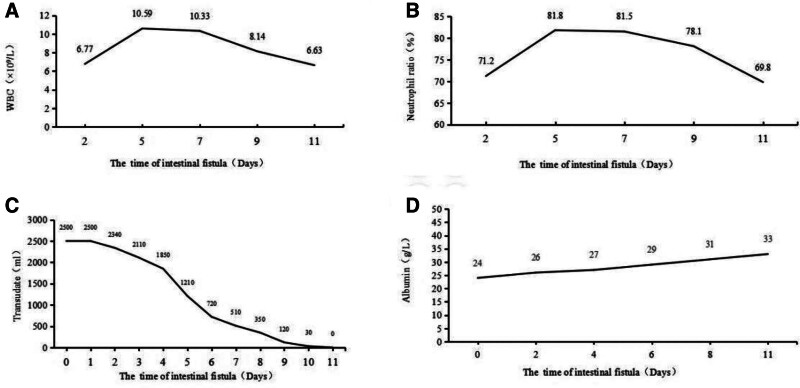
Changes in infection indicators, extravasation volume, and serum albumin. (A) Changes in WBC count. (B) Changes in the neutrophil ratio. (C) Changes in extravasation volume. (D) Changes in serum albumin. WBC = white blood cell.

**Figure 3.**
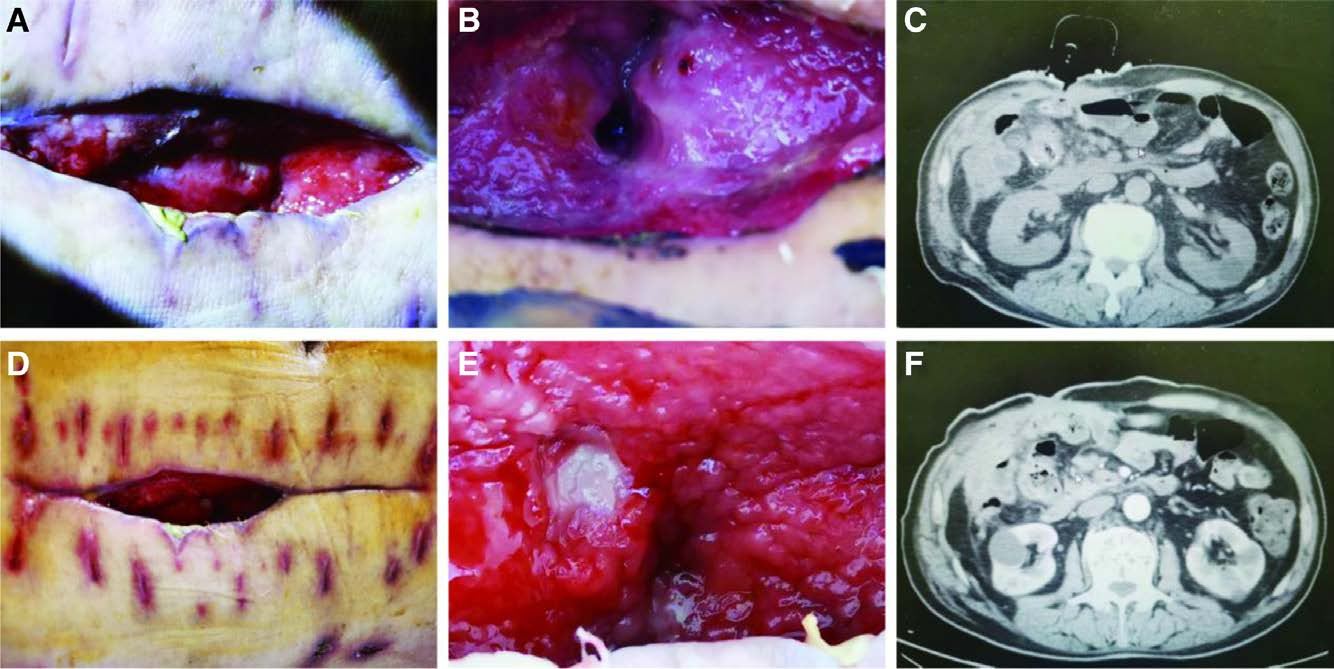
Incision, intestinal leakage, and CT after enterocutaneous fistula. (A) Necrotic tissue at the bottom of the incision was replaced with fresh granulation tissue. (B) The intestinal leakage was significantly narrower than the previous leakage. (C) Granulation tissue growth and narrowing of fistula tract. (D) Further narrowing of the incision. (E) Intestinal leakage healed after treatment and the incision was lined with fresh granulation tissue. (F) Intestinal leakage healed, and the fistula tract was completely filled with fresh granulation. CT = computed tomography.

After the occurrence of enterocutaneous fistula, routine blood tests showed an elevated white blood cell count and neutrophil ratio, decreased serum albumin, and a large volume of extravasation. Following treatment with a dome-shaped negative-pressure irrigation and drainage device, the white blood cell count and neutrophil ratio returned to normal levels, the extravasation volume gradually decreased to none, and the serum albumin gradually increased (Fig. 2). After 11 days of treatment with a dome-shaped negative-pressure irrigation and drainage device, the necrotic tissues in the incision were lysed and gradually replaced with fresh granulation tissues. The size of the incision was further reduced, and the fistula gradually narrowed until it was completely closed (Fig. 3). After the patient’s enterocutaneous fistula healed, their diet was gradually transitioned from liquid food to a normal diet. Reexaminations were conducted 30, 60, and 90 days after the fistula healing, which showed that the fistula had healed well without recurrence, and the patient was in good nutritional status.

### 2.4. Dome-type negative-pressure rinsing and drainage device

#### 2.4.1. Preparation of dome-shaped negative-pressure irrigation and drainage device

**Figure 4.**
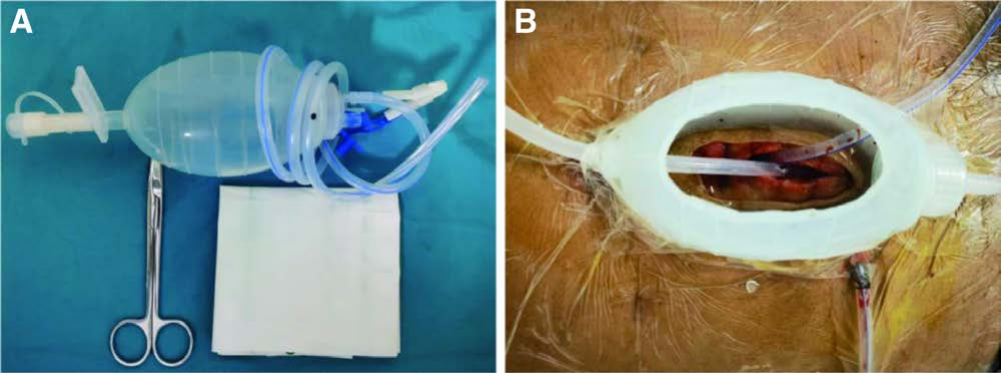
Dome-type negative-pressure rinsing and drainage device. (A) Items required for the device. (B) Device connection and usage.

Preparation of items: A single-use sterile drainage set (drain pot and drain tube), sterile breathable film, sterile gauze, stoma adhesive ring and gel, and sterile wire cutters (Fig. 4A). Preparation of the main body: The drainage pot was cut into longitudinal rows and the 2 ends of the connecting pipeline were retained. From the 2 ends of the pot body through the body of the pot into a porous pipeline as a rinse tube and drainage tube in the pot body at the top of the opening (suitable for the size of the incision). Device connection: the use of stoma adhesive ring and stoma glue to fix the pot body in the normal skin around the enterocutaneous fistula, and sterile breathable film paste the pot body edge and skin to enhance the fixation effect; in the pot at the top of the opening to adjust the flushing tube and drainage tube placed around the enterocutaneous fistula. The opening was then covered with sterile gauze; the irrigation tube was connected to saline irrigation, and the drainage tube was connected to negative-pressure suction; the irrigation and negative-pressure drainage were turned on at the same time, and the balance between the irrigation speed and negative-pressure suction was adjusted to achieve a balance (Fig. 4B).

Usage: when lying down, the original pipeline of the pot body is clamped shut, opening the flushing pipeline connected to saline flushing, opening the drainage pipeline connected to negative-pressure suction to realize the function of continuous flushing and drainage, and through the transparent pot to observe the fistula and granulation tissue to realize the visual function, and the pot body to realize the function of restricting the overflow of digestive fluid. When you are free to move, close the flushing and drainage lines, open the lower end of the pot to connect the drainage bag, and leak liquid into the drainage bag.

#### 2.4.2. Improvement of dome-shaped negative-pressure irrigation and drainage device

**Figure 5.**
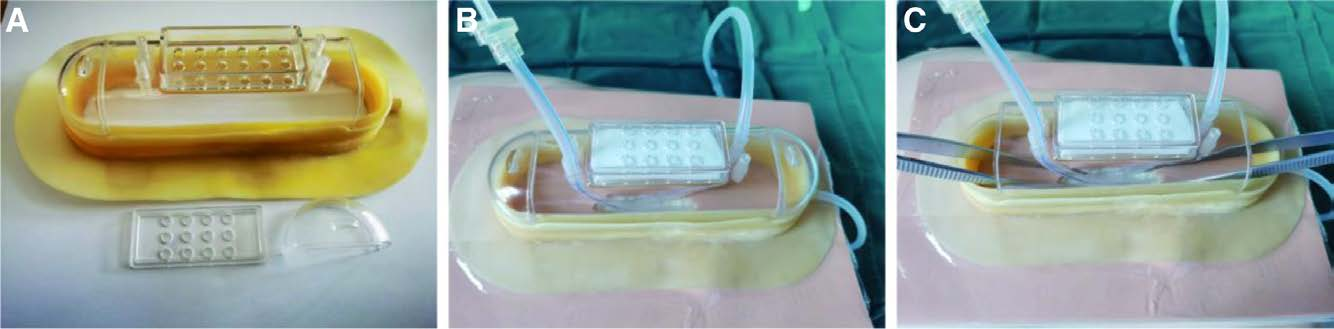
Improvement of dome-shaped negative-pressure irrigation and drainage devices. (A) Strip-shaped device used to treat enterocutaneous fistulas. 1:detachable chassis. 2: silicone base. 3: top cover. 4: operational hole cover. 5: bottom-annular drainage pipeline. 6: operation hole. (B) Flush diversion model. 1: flushing lines. 2: drain line. 3: bottom loop drainage path. 4: operation hole. (C) wound-dressing change mode of operation.

Based on the fact that the device made from the existing medical material could not present all the design advantages, a more perfect device was designed through improvement and a national utility model patent was obtained (patent no. 2022205366226). A groove was provided in the opening of the device to place gauze. A hollow removable sump was provided in the groove, and the bottom of the device was set up as a ring-shaped drainage tube (Fig. 5). Improvements have been made to make the device visual, maneuverable, flushable, drainable, and capable of intercepting overflow.

## 3. Discussion

Surgery is a common cause of enterocutaneous fistulae, which are pathological passages between the digestive tract and outside of the body. When an enterocutaneous fistula is formed, digestive fluids and feces overflow from the fistula, resulting in redness, swelling, erosion, pain, and secondary infection of the soft tissues surrounding the fistula.^[7]^ Previous studies on enterocutaneous fistula closure are very unlikely, according to reports of global enterocutaneous fistula closure rates of 19% to 92%^[8,9]^ adequate and effective drainage of the fistula is one of the important measures to promote fistula healing, but also needs to provide the patient with good nutritional support, control of infections, and correction of water electrolytes and acid-base balance imbalance to promote healing of the fistula.^[10]^

The existing negative-pressure wound therapy (NPWT) systems refer to a category of medical technology and equipment systems that establish a controlled negative-pressure environment in the wound or fistula area to achieve drainage, wound cleansing, and auxiliary healing. They are currently a common method for treating enterocutaneous fistulas.^[11,12]^. However, NPWT systems often experience negative-pressure seal failure due to issues such as irregular wound shapes, resulting in failure to drain in a timely manner. For instance, excessively high negative pressure may damage the fistula and wound tissues. Additionally, the lack of integrated irrigation in NPWT systems leads to the inability to dilute leaked fluid, which then corrodes the fistula and wound. Moreover, this absence of irrigation easily causes blockage of the drainage pathway, preventing adequate drainage and thus affecting the therapeutic effect. At the same time, the high cost of NPWT systems imposes an economic burden on patients.

The drip double trocar method,^[13]^ which is curable in most patients, with those who are not cured awaiting definitive surgery for enterocutaneous fistulas.^[14]^ The drip double trocar consists of an inner tube and anouter tube, which are coaxially nested. The inner tube (irrigation tube) continuously drips physiological saline to dilute the intestinal fluid leaking from the fistula, soften necrotic tissues and food residues. The outer tube (suction tube) is connected to a negative-pressure device to promptly suck out the diluted intestinal fluid, irrigation fluid, exfoliated tissues and pus from the body, preventing the accumulation of fluid around the fistula. Through the closed-loop mechanism of “irrigation – dilution – suction,” the drip double trocar method avoids the continuous stimulation of the fistula by intestinal fluid and necrotic tissues, creating conditions for the repair of the fistula. Xu et al found that the application of the modified drip double trocar method compared with the traditional method of dressing change in the treatment of enterocutaneous fistula results: drip double trocar method after treatment of enterocutaneous fistula healing time of 21.62 ± 5.20 days, the average number of daily dressing changes is 0.69 ± 0.21 days. The healing time of the fistula was 25.93 ± 4.23 days, and the average daily number of dressing changes was 2.62 ± 0.65 days after treatment with the traditional dressing change method.^[15]^ The drip double cannula method is obviously better than the traditional dressing change method, but still can’t solve the problem of leakage spillage and corrosion of the skin when the flow of the leakage fluid increases. Based on the therapeutic basis of the previous studies, we made a dome-shaped negative-pressure irrigation and drainage device by using the existing medical materials, and applied it to the clinical treatment of enterocutaneous fistula. The device was fixed on the normal skin surrounding the wound. The irrigation line was connected to physiological saline, and the suction line to negative pressure, thereby achieving a balance between irrigation and drainage. The dome served to prevent fluid from spilling out; meanwhile, due to its transparency, we observed the changes of the fistula and the freshness of the wound through the dome on a daily basis. We performed dressing changes on the wound once every 2 days through the operation hole. After treatment with this device, the healing time of the fistula in our patient was 11 days, and the patient’s pain was significantly reduced.

The healing cycle of the enterocutaneous fistula was shorter in patients treated with a dome-shaped negative-pressure irrigation and drainage device for several reasons: To avoid the corrosion of skin soft tissue caused by high-flow leakage of enterocutaneous fistula spillage and to produce a protective effect on the skin soft tissue around the fistula, which is conducive to the repair of trauma; to achieve high-flow flushing and dilute the leakage fluid to improve the drainage effect is better; more drainage tubes, a larger diameter, are not easily clogged, so the drainage effect is better; and can be calculated accurately to determine the amount of the patient’s leakage fluid and nutritional support.

This case report has several limitations. Firstly, it is based on only 1 specific male patient with underlying diseases, resulting in an extremely small sample size, which makes it impossible to verify the universality of the dome-shaped negative-pressure irrigation and drainage device for enterocutaneous fistulas of different types and etiologies. Secondly, the study lacks controls and is subject to potential confounding factors such as nutritional status. Thirdly, there is no control group, randomized design, or prospective planning, leading to a risk of bias and making it difficult to objectively demonstrate the advantages of this device over traditional treatments. Additionally, the potential safety risks and consumable costs of the device have not been systematically evaluated, and the independent therapeutic contribution of the device itself, as distinct from that of the basic treatment, has not been isolated.

## 4. Conclusion

This case demonstrates that the application of the dome-shaped negative-pressure irrigation and drainage device in the treatment of enterocutaneous fistulas is a promising method. Initial research has shown that using this device can promote fistula healing, protect damaged skin, stimulate granulation tissue growth, and reduce the frequency of dressing changes. The patient reported that both physical and psychological distress were alleviated during the treatment period. In the future, we will use this case as a pilot for a larger clinical series, further expanding the device’s application in enterocutaneous fistula cases to properly evaluate its efficacy, safety, and cost-effectiveness.

## Acknowledgments

I would like to thank Zhide Sun for providing me with many suggestions and advice. In addition, I am deeply grateful to my colleagues for their contributions to this thesis in various ways.

## Author contributions

**Conceptualization:** Jun Gao.

**Data curation:** Jun Gao, Hao Sun, Xiyan Qin.

**Resources:** Xiyan Qin.

**Writing – original draft:** Jun Gao.

**Writing – review & editing:** Zhide Sun.
